# Expression of Concern: Blockade of interleukin-17A protects against coxsackievirus B3-induced myocarditis by increasing COX-2/PGE2 production in the heart

**DOI:** 10.1093/femspd/ftaf010

**Published:** 2025-08-29

This is an expression of concern regarding: Yuquan Xie, Ruizhen Chen, Xian Zhang, Yong Yu, Yingzhen Yang, Yunzeng Zou, Junbo Ge, Haozhu Chen, Blockade of interleukin-17A protects against coxsackievirus B3-induced myocarditis by increasing COX-2/PGE2 production in the heart, *FEMS Immunology & Medical Microbiology*, Volume 64, Issue 3, April 2012, Pages 343–351, https://doi.org/10.1111/j.1574-695X.2011.00918.x

Concerns regarding similarities within Figure 5 were raised on Pub Peer [https://www.pubpeer.com/publications/5E4E2ED18184C407C8BA48E14E9A01#] and subsequently were flagged to the Editor-in-Chief in 2025.

The journal attempted to contact the corresponding author but has not received a response and is investigating our concerns about Figure 5 in line with COPE guidance. The journal is publishing this Expression of Concern to notify readers of the concerns whilst the outcome of the investigation is pending and advises readers to examine the details of this study with particular care.

